# How to Realize Business Model Innovation for New Ventures? Psychological Capital and Social Capital Perspective

**DOI:** 10.3389/fpsyg.2022.707282

**Published:** 2022-06-13

**Authors:** Jian Zhou, Yubo Liu, Peng Yang, Qinqin Cao

**Affiliations:** Department of Business Management, School of Business, Qingdao University, Qingdao, China

**Keywords:** business model innovation, psychological capital, social capital, resource conservation theory, signal theory, fsQCA

## Abstract

Business model innovation has become a necessary means for enterprises to break through path constraints, achieve sustainable development, and obtain sustainable competitiveness, which has been paid more and more attention by entrepreneurs and scholars. Based on the resource conversation theory and signal theory, this study constructs a research model with psychological capital (PC) and social capital (SC) as independent variables and business model innovation as dependent variables along the logical path of “resource acquisition-resource utilization.” By dividing business model innovation into pioneering business model innovation and perfect business model innovation, we use fuzzy-set qualitative comparative analysis (fsQCA) to explore the impact of dual capital on business model innovation of new ventures. This study takes entrepreneurs from the eastern part of China's seven entrepreneurship active provinces as investigation objects, based on the analysis of the collected 242 valid questionnaire results, emphasizing that any single antecedent can not be a sufficient and necessary condition for pioneering and perfect business model innovation. In this case, we carried out research with a certain antecedent variable as the core and supplemented with other antecedent variables to form seven different configurations. The results showed that the combination of the antecedent variables could effectively achieve the pioneering and perfect business model innovation. The theoretical contributions of this study are as follows: (1) it enriches the research on the antecedents of business model innovation in new ventures; (2) it expands the application scenarios of resource conversation theory and signal theory; and (3) it is emphasized that the innovation of business model of new ventures is the result of the interaction and value-added linkage of various internal and external resources.

## Introduction

In the digital Internet era with rapid economic development and rapid knowledge dissemination, continuous innovation has become an important way to ensure that enterprises gain sustainable competitiveness (Argyres et al., 2020; Shaheer and Li, 2020; Zhou et al., 2022a). In the current economic environment and market competition, the requirements for enterprise innovation have reached a new height. It is difficult for enterprises to win in the fierce industrial competition only by putting forward new ideas and developing new products through technological innovation (Chesbrough, 2017). In this situation, business model innovation has been paid more and more attention by new ventures, and business model innovation has become the key means for new ventures to break through path constraints, realize disruptive innovation, and catch up with others (Pedersen et al., 2018; Haaker et al., 2021). For example, the online car-hailing service model proposed by Didi Taxi has completely broken the traditional car-hailing mode, and has been favored by people, thus becoming a mainstream car-hailing model. The failures of large companies, such as Nokia and Myspace, also show the importance of enterprises adapting to the new market environments through business model innovation, so as to achieve sustainable development of enterprises. Zott and Amit (2008) pointed out in their research that a business model is an important way for enterprises to create and utilize value, it can reflect the strategic choice of the enterprise to some extent, and the business model matches the strategic choice of enterprises to achieve value creation. With the wider and deeper application of the Internet in various industries, technologies, such as big data, blockchain, and artificial intelligence (AI), give enterprises more innovative choices and opportunities for business model innovation (Jetter et al., 2009; Yang and Han, 2019), emerging business models can bring remarkable performance to enterprises, and sustainable competitiveness is born under such conditions (Teece, 2009; Foss and Saebi, 2017). However, for new ventures, there are only a few who succeed through business model innovation, because new ventures are faced with many difficulties in the initial stage of starting a business, and resource constraint is one of the most important difficulties, which can be described as “a clever woman cannot cook meals without rice” (Gegenhuber and Dobusch, 2017; Lundmark et al., 2020). Since the 1990s, business model research has become the focus of scholars' attention (Chesbrough, 2010; Baden-Fuller and Haefliger, 2013), the research on new ventures has just begun to rise in recent years. The scholars of new ventures start their research from the perspectives of new knowledge (Arfi and Hikkerova, 2019), resource action (Cui and Pan, 2015), entrepreneurs' previous experience (Martins et al., 2015), and new ventures' management ability (Futterer et al., 2018). A lack of key resources, such as knowledge, experience, skills, and talents, is the dilemma that new ventures must face directly, which is also the main reason for the failure of business model innovation of new ventures. Therefore, it is meaningful to study from the perspective of “resource acquisition-resource utilization” to help new ventures overcome the dilemma of “no rice,” successfully implement business model innovation, and achieve sustainable development.

New ventures mainly acquire resources in the traditional way by investing in tangible capital, but pay less attention to intangible capital (Hanlon and Saunders, 2007; Frid, 2014). With the rapid development of the economy and the increasing uncertainty of the market environment, researchers gradually realize that entrepreneurs' inherent intangible capital, such as social capital (SC) and psychological capital (PC), has a far-reaching impact on the growth and development of enterprises (Prashantham and Dhanaraj, 2010; Bizri, 2017; Bockorny and Youssef-Morgan, 2019). Obtaining resources are of higher quality and more difficult for competitors to imitate through investing in intangible capital, which can enable enterprises to have sustainable competitive advantages (Baluku et al., 2018). Therefore, it is of great significance to study how new ventures use intangible capital to obtain resources and how intangible capital influences new ventures' business model innovation and further influences the growth and development of new ventures. Zou et al. (2016) pointed out that with the increasingly fierce market competition and the accelerating speed of technological innovation, researchers and entrepreneurs have deeply realized that it is far from enough to keep the sustained growth of enterprise performance when only relying on tangible and easily imitated economic capital, such as large amounts of funds, equipment, and technology in the traditional sense. Under the current market environment, the researchers must pay attention to entrepreneurs' inherent intangible capital, such as SC and PC (Bockorny and Youssef-Morgan, 2019). With the improvement of consciousness, entrepreneurs began to pay more attention to intangible capital, including SC and PC (Luthans et al., 2004; Bockorny and Youssef-Morgan, 2019). SC can be understood as the vertical connection, horizontal connection, and social connection between actors and other actors or organizations, and the ability to obtain rare resources through this connection (Khan et al., 2020). With the increase of market openness and transparency, and the enhancement of competitiveness, it is difficult for enterprises to stand out in the fierce industry competition only by their own internal resources and technology, which requires enterprises to broaden their social network relations, increase cooperation with other stakeholders, and can even cross industries to seek partners. The signal theory emphasizes that sending positive signals to other stakeholders in the SC structure through the signaling process can promote the cooperative relationship between enterprises and stakeholders (Connelly et al., 2011). From the perspective of the individual, the innovation process is social, and the more SC an individual has, the more opportunities he will have to carry out innovative activities (Deng et al., 2020). For new ventures, the SC of entrepreneurs is very important for the development of enterprises. In the new period of lack of experience, resources, and technology, it is the most convenient and effective way for enterprises to quickly enhance their strength by drawing resources through social network relations. Entrepreneurs' own SC scale is also closely related to the success rate of entrepreneurship (Buttice et al., 2017). Entrepreneur's SC is based on the perfection and level of entrepreneur's PC. Compared with SC, PC will affect the success rate of entrepreneurship from the long-term development of enterprises (Contreras et al., 2017). PC refers to a positive psychological state in the process of individual growth and development, which affects individual development as an internal factor, and the PC of entrepreneurs or managers will inevitably penetrate into the management methods and strategic choices of enterprises, thus having an impact on the development of enterprises (Hasan et al., 2019; Tang, 2020). According to the resource conservation theory, the self-efficacy, hope, optimism, and resilience of PC are personal resources, and entrepreneurs must invest these resources to prevent the damage of resources and obtain more resources (Hobfoll et al., 2018). Among individual intangible capital, PC is the core element, and the perfection and level of PC have a deeper influence on individuals.

On the basis of summarizing the relevant research, this article takes the business model as the research object and constructs a research model of the influence of two capitals on business model innovation from the perspective of entrepreneurs' SC and PC. As SC, PC, and business model innovation have nonlinear relationship with multiple conditions, which is the result of multiple factors, the existing research pays too much attention to the linear relationship between the factors and the explanation of the mediation effect mechanism and lacks in-depth exploration to enhance its multiple concurrent paths (Guo et al., 2013; Zhang et al., 2016). This study uses fuzzy-set qualitative comparative analysis (fsQCA) to o?er a new perspective on the configuration of multiple capital antecedents that drive business model innovation. Instead of a one-size-fits-all approach, we used a more comprehensive set of options with business model innovation for new ventures and enriched the research of SC and PC on enterprise growth. At the same time, this study can remind entrepreneurs and managers to pay enough attention to individual intangible capital and cultivate and improve the composition and scale of the two capitals, which has certain research value in both theoretical and practical research.

## Theory and Research Framework

### Business Model Innovation

In recent years, more scholars have paid attention to the study of business model (Teece, 2009; Chesbrough, 2010; Foss and Saebi, 2017). By sorting out relevant literatures, it is found that scholars mainly study and expound the connotation of business model from the perspectives of value creation, resource capability, and stakeholders (Wirtz et al., 2016). As for the definition of a business model, the theoretical circles have not reached a consensus so far, and many scholars have put forward their own different opinions. Among them, the concepts put forward by Amit and Zott (2001) have been widely recognized by scholars, who believe that a business model is a cross-border transaction system built by enterprises around stakeholders, including three elements, namely, transaction structure, transaction content, and transaction governance. Business model innovation refers to the revision and change of the original business model (Cavalcante et al., 2011), which means that the old resource model is broken and the new resource model is built (Brea-Solís et al., 2015). Teece (2009) directly points out that the change in business model is supplemented and optimized for enterprise resources. Business model innovation can help enterprises gain sustainable competitive advantage and seek long-term development, but the key is that only successful business model innovation can achieve this one. There are a few cases of failure in business model innovation, especially in the initial stage of start-ups, where there are various disadvantages, such as lack of resources, experience, technology, and talents, so it is very difficult to achieve breakthroughs through business model innovation. Clauss (2017) pointed out that the value logic of business model emphasizes the value acquisition in value creation, while the value potential of business model innovation is conditional, and the initial start-ups must use the corresponding resource structure to effectively tap the value. Therefore, it is very important for business model innovation activities for entrepreneurs and managers to decide how to obtain resources, what resources to obtain, where to obtain them, and how to reconstruct and allocate these resources. Arfi and Hikkerova (2019) believed that new knowledge is an important driving force for new ventures to achieve business model innovation, and from the perspective of new knowledge acquisition, it is found that there are two ways to acquire new knowledge, namely, external knowledge search and internal knowledge creation, which can effectively promote the business model innovation of new ventures. Sosna et al. (2010) studied the impact of entrepreneurial learning behavior on business model innovation of Spanish dietary companies from the perspective of trial and error learning. The results show that enterprises can acquire and accumulate knowledge and skills through entrepreneurial learning, improve the innovation ability of enterprises, and thus positively promote the business model innovation of new ventures.

Many scholars have studied the business model innovation mechanism from the perspectives of resource acquisition, resource utilization, and resource integration, and believe that improving the resource scarcity dilemma of new ventures is of great significance to the business model innovation of new ventures (Foss and Saebi, 2017; Lopez et al., 2019). Therefore, this article studies the influence mechanism of entrepreneurs' intangible capital on business model innovation of new ventures from the perspective of new resource accumulation of new ventures, that is, the PC and SC of entrepreneurs.

### Psychological Capital

The PC is defined as the psychological state that employees show during their own growth, which can lead to employees' active organizational behavior (Luthans and Youssef, 2004). Entrepreneurs/PC is an important variable that affects their individual behavior ability. Based on the research of Luthans and other scholars, this article divides the structural elements of PC into four dimensions, namely, self-efficacy, optimism, hope, and resilience (Luthans et al., 2004, 2005; Baron et al., 2016). The four dimensions of entrepreneurs' PC have a strong correlation and emphasis. Entrepreneurial self-efficacy refers to the individual's belief in stimulating motivation, mobilizing cognitive resources, taking action to complete a specific job, and emphasizing the self-cognitive ability of entrepreneurs. Entrepreneurial optimism and entrepreneurial hope refer to a positive state of motivation and attach importance to the positive emotion of attribution mode when facing challenges and failures. Psychological resilience refers to a positive psychological state that can quickly rebound or recover from adversity, conflict, and failure, with an emphasis on persisting in goals.

What kind of psychological state can help entrepreneurs better undertake their role? At present, scholars not only pay attention to the objective problems encountered in the entrepreneurial process but also pay more attention to the study of entrepreneurs' own subjective will and cognitive consciousness (Oh et al., 2014; Zhu et al., 2018). Luthans et al. (2004) thought that PC is an individual's positive psychological ability composed of many factors, which has a significant impact on individual's cognitive process, job satisfaction, and performance. Employees with higher self-efficacy are more willing to face challenging jobs, and PC can help employees generate new ideas and show more innovation. Second, the research shows that PC is helpful to predict individual high-performance work and happy work index, and is beneficial to realize positive organizational behavior (Avolio et al., 2004; Gielnik et al., 2020). Entrepreneurs with high entrepreneurial hope and optimism will keep a positive attitude in the face of a complex entrepreneurial environment and will have a better chance to find solutions to business challenges and innovate business models (Hmieleski and Baron, 2009; Lee and Na, 2013; Fourati and Attitalah, 2018). In addition, Li et al. (2019) analyzed the impact of PC on the creativity of leaders and employees based on the four dimensions of self-efficacy, hope, optimism, and resilience, and the scholar found that “hope” and “self-efficacy” in PC can promote entrepreneurs' relevant awareness and characteristics, while “optimism” and “resilience” can improve entrepreneurs' entrepreneurial knowledge and ability. There are also documents that have studied the mechanism behind employee's PC and high-performance work systems, explaining the direct effects of employee's PC on high-performance work systems at the organizational level (Miao et al., 2020). Therefore, PC as a positive psychological state of entrepreneurs can create a new situation by using the existing positive state, shape the core competitive advantages of entrepreneurs in knowledge and skills, cultivate entrepreneurs' excellent psychological quality, personality traits, and entrepreneurial ability, influence entrepreneurs' individual behavior ability, and penetrate into the strategic choice and management methods of enterprises, which will drive entrepreneurs to change their business model cognition in time, and then drive business model innovation.

Based on the abovementioned discussion, we believe that entrepreneurs' PC is helpful to promote the business model innovation of new ventures.

### Social Capital

The SC is the connection between actors and society and the ability to access scarce resources through this connection. According to Nahapiet and Ghoshal (1998), this article divides SC into three dimensions, namely, cognition, relationship, and structure. The cognitive dimension mainly includes the common cognition of entrepreneurs and the methods and means of common narrative; the relationship dimension mainly includes the family and social relationship network of entrepreneurs; the structural dimension mainly refers to the scale of information and knowledge that entrepreneurs can obtain in financing and entrepreneurship (Nahapiet and Ghoshal, 1998).

A large number of researchers have studied the endogenous mechanism of SC's influence on innovation (Mazzucchelli et al., 2019; Yeşil and Dogan, 2019; Hasan et al., 2020). First, from the perspective of cognition, entrepreneurs optimize the resource allocation of enterprises by using SC to communicate and collaborate across organizations and promote the rationalization of the flow of production factors of enterprises (Deng et al., 2020). Second, from the perspective of relationship, SC can promote individuals to obtain resources and relationship networks owned by groups. Scholars, such as Maurer and Ebers (2006), believe that the network resources embedded by entrepreneurs have a significant effect on the innovation of the opportunities they identify. Entrepreneurs can identify the upgrading and optimization of the interaction between the elements of the business model through the social network, and make reasonable strategic adjustments. Finally, from the perspective of structure, when entrepreneurs use SC to conduct political and commercial business relations, they can, to a certain extent, dig for more financing and entrepreneurial information to identify business model innovation opportunities (Carmona-Lavado et al., 2010). The process of business model has certain sociality. When individuals and enterprises have more social resources, they have more opportunities to innovate and change the business model (Madhavaram and Hunt, 2017). SC is beneficial for enterprises to acquire and utilize the existing resources and tap new resources, which can effectively improve enterprises' ability to integrate entrepreneurial resources and provide a material basis for business model innovation. The social connection between enterprises and government departments, enterprises and enterprises, and enterprises and managers is beneficial to enterprises to dig out key information, reasonably promote effective social interaction between enterprises inside and outside, identify business model innovation opportunities, and innovate business models from the perspectives of stakeholders, enterprise strategy, and resource integration (Neira et al., 2017; Khazami et al., 2020).

Therefore, we infer that SC is helpful to promote the business model innovation of new ventures.

**Figure 1:**
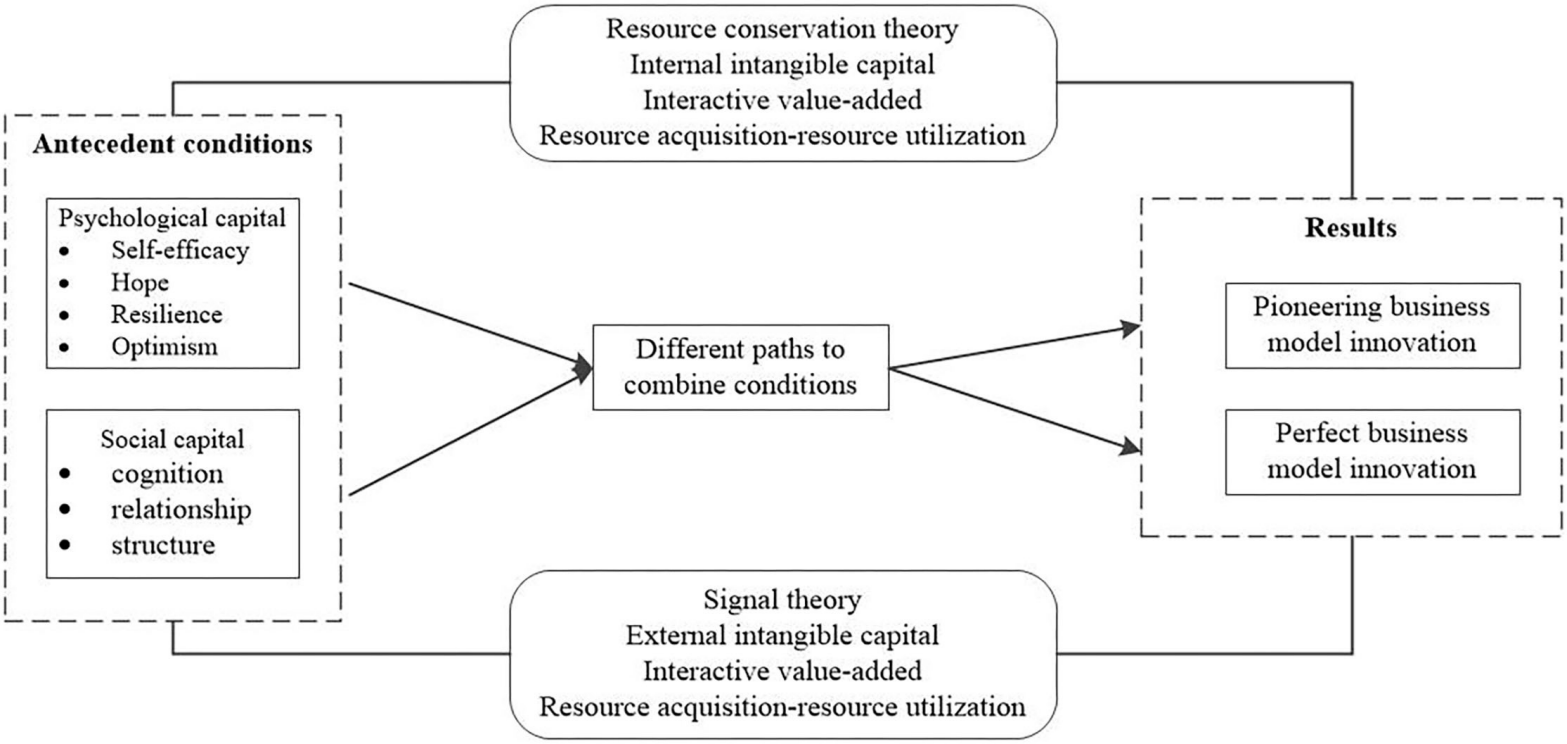
Research framework.

Business model innovation of new ventures is the result of multiple factors. Based on the resource conservation theory, signal theory, and “resource acquisition-resource utilization” logic, this study holds that different dimensions of entrepreneurial PC and SC can produce an interactive value-added effect, and forms different combination paths to act on the business model innovation process. Therefore, this article proposes a research framework, as shown in Figure 1.

## Method and Design

### Research Methods

In this article, the fsQCA method is used to analyze the obtained research data. The QCA method originated from the field of sociological research and adopted the core ideas of set theory and Boolean operation, which can test the complex causality caused by the interaction and influence of multiple reasons, and explore the concurrency logic configuration of different paths leading to the same goal (Fiss, 2007, 2011; Du and Kim, 2021). As stated earlier, the complexity and unpredictability of business model innovation of new ventures can't be explained by a single variable and single channel, and the multidimensional nature of entrepreneurial PC and SC makes the dimensions of entrepreneurial PC and SC interact with each other and acts together in the process of business model innovation of new ventures. Therefore, it is appropriate and necessary to choose fsQCA which can handle continuous data to analyze the configuration that leads to business model innovation.

### Sample and Data Collection

The main research content of this article involves entrepreneurs' dual capital and business model innovation of new ventures. Due to the secrecy of dual capital and the difficulty of evaluating business model innovation, it is difficult for ordinary entrepreneurs to complete the data. Therefore, this article focuses on entrepreneurs who participate in the daily operation of new ventures. In the actual distribution process of questionnaires, electronic questionnaires, mailing questionnaires, and on-site questionnaires were adopted in parallel. The questionnaires were collected from May to June 2019, covering seven provinces in eastern China with active entrepreneurial activity. A total of 500 questionnaires were sent out in three ways, including 350 electronic questionnaires and 150 questionnaires, sent by mail and on-site. Through the confirmation of the three ways, 242 valid questionnaires were collected, and the overall effective recovery rate was 48.4%. The characteristics of questionnaire data are shown in Table 1.

**Table 1:** Basic information of questionnaire (*n* = 242).

		**Number**	**Proportion**
**Demographic characteristics of entrepreneurs**			
Gender	Male	168	69.4%
	Female	74	30.6%
Education level	Junior high school and below	3	1.2%
	High school	4	1.7%
	Secondary specialized school	19	7.9%
	Bachelor's degree	170	70.2%
	Graduate degree	46	19.0%
Age	<30	113	46.7%
	31–40	116	47.9%
	41–50	10	4.1%
	>50	3	1.2%
**Enterprise characteristics**			
Enterprise age	<1	22	9.1%
	2–3	92	38.0%
	4–5	54	22.3%
	6–8	74	30.6%
Number of employees	<10	27	11.2%
	10–50	86	35.5%
	51–100	90	37.2%
	>100	39	16.1%

### Measuring Tools and Calibration

In the fsQCA, this study divides the business model innovation of new ventures into pioneering business model innovation and perfect business model innovation. The antecedents include self-efficacy, hope, resilience, and optimism in entrepreneurial PC, and cognition, relationship, and structure in SC.

#### The Result Variable

Business model innovation of new ventures is divided into two types, namely, pioneering business model innovation and perfect business model innovation.

The pioneering business model innovation mainly draws on the innovative business model of Zott and Amit (2007), the strategic business model innovation of Aspara et al. (2010), the proactive market orientation of Narver et al. (2004), the destructive innovation of Christensen (2006), the radical innovation capability of Subramaniam and Youndt (2005), the exploratory business model transformation of Osiyevskyy and Dewald (2015), and the strategic research thinking and rational core of exploratory innovation of He and Wong (2004). The perfect business model innovation mainly draws on the research ideas and reasonable cores, such as Zott and Amit's (2007) efficient business model, Narver et al.'s (2004) responsive market orientation, Subramaniam and Youndt's (2005) progressive innovation capability, Osiyevskyy and Dewald's (2015) utilizing business model transformation, and He and Wong's (2004) utilizing innovation strategy. Among them, pioneering business model innovation includes 8 items: typical items include “discovering new opportunities and opening up new markets in an unusual way,” and perfect business model innovation includes 8 items: typical items include “in terms of market development, we tend to follow the innovation of the market leader.”

#### Antecedents

Entrepreneurial PC is divided into four dimensions, namely, self-efficacy, hope, resilience, and optimism based on the research results of Luthans et al. (2007), and a 24-item scale was used for measurement, with six items per dimension. Typical items of self-efficacy include “I believe I can analyze long-term problems and find solutions,” typical items of hope include “If I find myself in trouble at work, I can come up with many ways to get rid of it,” typical items of resilience include “I usually take the pressure at work calmly,” and typical items of optimism include “When I encounter uncertain things at work, I usually look forward to the best results.”

Entrepreneurial SC, referring to the research results of Nahapiet and Ghoshal (1998), is measured with a 10-item scale, which mainly includes entrepreneurs' cognition, relationship, and structure. The cognitive dimension consists of 4 items and typical items include “successful entrepreneurs will attract many people's attention and admiration,” the relational dimension consists of 4 items and typical items include “young people are encouraged to start their own businesses independently,” and the structural dimension consists of 2 items and typical items include “when I have difficulties, there will be many friends and relatives to help me.”

The fsQCA method adopted in this article can express cases, such as “completely subordinate” and “completely non-subordinate,” and fuzzy sets are suitable for dealing with continuous variables. The 7-point Likert-type scale is selected, in which “7” means ‘complete membership” in this study. However, the distribution of questionnaire data is characterized by agglomeration; therefore, “4” can not be used for demarcation. Instead, the average value of questionnaire actual data is used as the demarcation line between complete membership and complete non-membership, that is, the anchor point, “1,” is used to indicate “complete non-membership.” Therefore, three thresholds are set, fsQCA3.0 is used to calibrate the data, and its value is converted into a 0–1 membership value. The evaluation criteria of related variables in this study are shown in Table 2.

**Table 2:** Assignment criteria (*n* = 242).

**Concept**	**Thresholds**		
	**Complete non-membership**	**Intersection**	**Complete membership**
Self-efficacy	1	5.45	7
Hope	1	5.18	7
Resilience	1	5.16	7
Optimism	1	5.23	7
Cognitive dimension	1	5.14	7
Relationship dimension	1	4.94	7
Structural dimension	1	4.45	7
Pioneering-BMI	1	5.14	7
Perfect-BMI	1	5.31	7

### Descriptive Statistics and Reliability and Validity Analysis

Descriptive statistical analysis and reliability and validity analysis of main variables involved in this article are listed in Tables 3, 4, respectively. Descriptive statistical analysis in Table 3 shows that the four dimensions of entrepreneurial PC are related to pioneering business model innovation (*r* = 0.39, *p* < 0.01; *r* = 0.45, *p* < 0.01; *r* = 0.42, *p* < 0.01; *r* = 0.41, *p* < 0.01) and perfect business model innovation (*r* = 0.53, *p* < 0.01; *r* = 0.53, *p* < 0.01; *r* = 0.51, *p* < 0.01; *r* = 0.46, *p* < 0.01). Three dimensions of SC and pioneering business model innovation (*r* = 0.34, *p* < 0.01; *r* = 0.40, *p* < 0.01; *R* = 0.17, *p* < 0.01) and perfect business model innovation (*r* = 0.38, *p* < 0.01; *r* = 0.33, *p* < 0.01; *R* = 0.14, *p* < 0.01) are also significantly positively correlated.

**Table 3:** Descriptive statistical analysis.

**Variable**	**1**	**2**	**3**	**4**	**5**	**6**	**7**	**8**	**9**
1. Self-efficacy	1								
2. Hope	0.48^**^	1							
3. Resilience	0.51^**^	0.51^**^	1						
4. Optimism	0.45^**^	0.48^**^	0.46^**^	1					
5.Cognitive dimension	0.40^**^	0.28^**^	0.33^**^	0.38^**^	1				
6.Relationship dimension	0.20^**^	0.24^**^	0.20^**^	0.28^**^	0.22^**^	1			
7. Structural dimension	0.12^**^	0.17^**^	0.15^**^	0.18^**^	0.25^**^	0.17^**^	1		
8. Pioneering BMI	0.39^**^	0.45^**^	0.42^**^	0.41^**^	0.34^**^	0.40^**^	0.17^**^	1	
9. Perfect BMI	0.53^**^	0.53^**^	0.51^**^	0.46^**^	0.38^**^	0.33^**^	0.14^**^	0.55^**^	1
Average value (m)	5.45	5.18	5.16	5.23	5.14	4.94	4.45	5.14	5.31
Standard deviation (SD)	0.85	0.84	0.85	0.87	0.94	0.99	1.32	0.81	0.79

*n = 242*,

** P < 0.01, *P < 0.05.

**Table 4:** Reliability and validity analysis (*n* = 242).

**Variable**	**Dimension**	**Item**	**Factor load**	**Cronbach's α**	**Variable**	**Dimension**	**Item**	**Factor load**	**Cronbach's α**
SC	CD	SC1	0.625	0.703	PC	SE	PC1	0.897	0.725
		SC2	0.842				PC2	0.853	
		SC3	0.943				PC3	0.801	
		SC4	0.823				PC4	0.819	
	RD	SC5	0.718	0.715			PC5	0.792	
		SC6	0.759				PC6	0.857	
		SC7	0.863			Hope	PC7	0.758	0.755
		SC8	0.777				PC8	0.840	
	SD	SC9	0.777	0.710			PC9	0.854	
		SC10	0.774				PC10	0.753	
BMI	Pioneering BMI	PBMI1	0.643	0.758			PC11	0.778	
		PBMI2	0.531				PC12	0.787	
		PBMI3	0.710			RES	PC13	0.815	0.763
		PBMI4	0.779				PC14	0.825	
		PBMI5	0.758				PC15	0.710	
		PBMI6	0.660				PC16	0.858	
		PBMI7	0.739				PC17	0.875	
		PBMI8	0.650				PC18	0.689	
	Perfect BMI	IBMI1	0.644	0.726		OPT	PC19	0.715	0.777
		IBMI2	0.779				PC20	0.729	
		IBMI3	0.778				PC21	0.778	
		IBMI4	0.670				PC22	0.799	
		IBMI5	0.790				PC23	0.816	
		IBMI6	0.739				PC24	0.907	
		IBMI7	0.683						
		IBMI8	0.788						

The results of reliability and validity analysis in Table 4 show that the reliability of Cronbach's α of the main variables involved in this article is >0.7, and the factor loads of other variables are >0.65, except for the pioneering business model innovation, which all meet the expected standards, indicating that the data collected in this article are suitable and can be analyzed in the next step.

### Analysis of Homologous Variance

As all the main variables in this article are answered by entrepreneurs according to the actual situation, there may be a problem of common method deviation. To solve this problem, in the variable measurement program, multiple items are used to measure different constructs. In the specific analysis, this article uses Harman's single-factor variance test to conduct an unrotated factor analysis on all items of the questionnaire. The results show that the variance explained by the first principal component is 25.78%, which does not account for half of the total variance explanation (62.37%), indicating that the common method deviation has less influence on the research results (Podsakoff et al., 2003).

## Data Analysis Results

### The Necessity and Sufficiency Test of Conditional Variables

The fsQCA method is used to test the necessary and sufficient conditions of whether each single antecedent variable involved in this article is a result variable, and the results are shown in Table 5. It can be seen that the result variables, whether pioneering business model innovation or novel business model innovation, are <0.9, and it can be considered that no single antecedent variable has become a sufficient and necessary condition that can lead to pioneering business model innovation and novel business model innovation.

**Table 5:** Necessary conditions and adequacy tests of conditional variables (*n* = 242).

**Antecedent condition**	**Pioneering innovation**		**Novel innovation**	
	**Adequacy consistency rate**	**Necessary coverage rate**	**Adequacy consistency rate**	**Necessary coverage rate**
SE	0.738911	0.895569	0.805215	0.882325
~ SE	0.852119	0.541154	0.836814	0.475157
Hope	0.834239	0.859455	0.889957	0.819764
~ Hope	0.809641	0.570196	0.800464	0.504035
Res	0.841450	0.860665	0.885538	0.809841
~ Res	0.811320	0.574215	0.794719	0.502901
OPT	0.805295	0.859825	0.854160	0.815420
~ OPT	0.814383	0.560092	0.816926	0.502344
CD	0.821298	0.827428	0.858468	0.773288
~ CD	0.806974	0.577233	0.811513	0.519008
RD	0.871777	0.796264	0.891283	0.727871
~ RD	0.760940	0.587253	0.781903	0.539529
SD	0.889361	0.682097	0.901779	0.618380
~ SD	0.692285	0.637033	0.707767	0.582310

### Precondition Configuration of Pioneering Business Model Innovation

The above analysis results of single variables show that the explanation of pioneering business model innovation by single antecedent variables is weak. To obtain the antecedent variable combination of pioneering business model innovation, this article puts seven antecedents into the fsQCA3.0 framework, and tries to analyze and explore the antecedent configuration that determines the pioneering business model innovation of new ventures. In the process of QCA analysis, the consistency threshold is set at 0.8, and the number of acceptable cases is set at 1. The data results show complex solutions and simplified solutions, and the intermediate solution is obtained on the basis of theoretical analysis of each antecedent variable. The specific results are shown in Table 6. Among them, symbol • or · represents the existence condition, symbol ⊗ or ⊗ represents the nonexistence condition, and “Blank” indicates that the existence or nonexistence of the condition in the configuration has no significant influence on the result. Meanwhile, symbol • or ⊗ represents the core causality condition and symbol • or ⊗ represents the auxiliary causality condition. After the analysis is completed, it is classified and integrated according to the exploration results. From the analysis of the results in Table 6, four main trigger modes can be found, and their overall consistency is 0.855472, and the overall coverage rate is 0.927492.

**Table 6:** Pre-condition configuration of perfect business model innovation.

**Configuration**	**Pioneering business model innovation**			
	**S1**	**S2**	**S3a**	**S3b**
SE	•	⊗	•	
HOPE	˙	˙		˙
RES	⊗	•	•	
OPT	⊗			
CD	˙		˙	⊗
RD		•	•	•
SD	•	•		
Consistency	0.863771	0.895278	0.892710	0.866858
Coverage rate	0.012447	0.000296	0.007902	0.007902
Net coverage rate	0.774177	0.933106	0.957455	0.957455
Overall consistency	0.855472			
Overall coverage rate	0.927492			

The specific results are as follows.

#### SE•HOPE•~OPT•CD•SD

Configuration S1 indicates that the core conditions that lead to pioneering business model innovation are self-efficacy in entrepreneurial PC and structural dimension in SC, supplemented by hope and cognitive conditions. Under this configuration, pioneering business model innovation can be realized even without resilience and optimism. High self-efficacy promotes entrepreneurs' belief in pioneering business model innovation, while the structural dimension makes entrepreneurs' information and knowledge scale adapt to this innovation situation, which helps new ventures to form the “unbalanced” first-mover advantage of the market and form new business model types with the help of hope and common cognition. Through the above analysis, this configuration is named “belief-oriented.”

#### ~SE•HOPE•RES•RD•SD

Configuration S2 shows that the core conditions leading to pioneering business model innovation are the resilience in entrepreneurial PC and the relationship and structural dimension in SC, supplemented by the hope condition. Under this configuration, pioneering business model innovation can be realized even if self-efficacy is lacking. SC can import information, knowledge and other resources through its relationship structure and network, and can integrate resources by embedding them. Due to the high risk of involvement in pioneering innovation, entrepreneurs are required to have the resilience to overcome difficulties and carry out continuous innovation, and realize pioneering business model innovation under the joint action of resources and resilience. Through the above analysis, this configuration is named “resilience-oriented.”

#### SE•RES•RD and SE•~HOPE•RES•~CD•RD

Configurations S3a and S3b indicate that the core conditions of pioneering business model innovation are self-efficacy and resilience in entrepreneurial PC and the relationship dimension in SC. Since the goal of pioneering business model innovation is to reconstruct the business transaction model and provide new transaction rules, which makes it highly innovative and leading, and at the same time makes this business model innovation face high risks, entrepreneurs are required to have high self-efficacy and resilience and provide resources through family and social relationship networks. In addition, the auxiliary condition of configuration S3a is the cognitive dimension in SC, and the auxiliary condition of configuration S3b is hope and lack of cognition, which indicates that entrepreneurs need common cognition to achieve pioneering business model innovation. If there is a lack of cognition, hope in entrepreneurial PC constitutes a complementary condition. Through the above analysis, this configuration is named “compound oriented type I.”

### Precondition Configuration of Perfect Business Model Innovation

The analysis results of a single variable also show that the explanation of perfect business model innovation is weak, this article brings seven antecedents into the fsQCA3.0 framework and identifies the antecedent configuration that determines the perfect business model innovation of new ventures, and the specific results are shown in Table 7. Four main trigger modes can be found by analyzing the results in Table 7, their overall consistency is 0.815955, and the overall coverage rate is 0.93404, specifically as follows.

**Table 7:** Pre-condition configuration of pioneering business model innovation.

**Configuration**	**Perfect business model innovation**			
	**S4**	**S5**	**S6**	**S7**
Self-efficacy(SE)	⊗	•	•
Hope(HOPE)	•	⊗		•
Resilience(RES)		˙	⊗	
Optimism(OPT)	•	•	•	•
Cognitive dimension(CD)			•	
Relationship dimension(RD)	•		•
Structural dimension(SD)	˙	⊗		˙
Consistency	0.838162	0.814640	0.893979	0.849984
Coverage rate	0.006347	0.012101	0.000110	0.007181
Net coverage rate	0.932365	0.943679	0.949660	0.967758
Overall consistency	0.815955
Overall coverage rate	0.934040

#### ~SE•HOPE•OPT•RD•SD

Configuration S4 shows that the core conditions leading to perfect business model innovation are hope, optimism in entrepreneurial PC, and relationship dimension in SC, supplemented by structural dimension in SC. The hope and optimism of entrepreneurial PC make entrepreneurs more able to face challenges and be courageous in making decisions and can overcome and resist the uncertainty in the process of business model innovation (Song and Song, 2021). The relationship and structural dimensions of SC support entrepreneurs' decisions from the level of resource supply on the basis of expanding the relationship network, which makes entrepreneurs make decisions that are conducive to business model innovation (Lofthouse and Storr, 2021). In addition, this configuration also shows that a perfect business model can be achieved without self-efficacy on the basis of the abovementioned core conditions. Through the above analysis, this configuration is named “Hope-oriented.”

#### SE•~HOPE•RES•OPT•RD•~SD

Configuration S5 shows that the core conditions leading to perfect business model innovation are self-efficacy in entrepreneurial PC, optimism, and relationship dimension in SC, supplemented by resilience in PC. Compared with configuration S4, the core conditions of this configuration are replaced by self-efficacy, and other core conditions are consistent, which indicates that self-efficacy and hope in entrepreneurial PC have a substitution effect on the basis of optimism and relationship dimension, and having one of the two can achieve perfect business model innovation. In addition, this configuration also shows the important value of resilience in improving entrepreneurial ability and competence to achieve business model innovation. Through the above analysis, this configuration is named “confidence-oriented.”

#### SE•~RES•OPT•CD

Configuration S6 shows that the core conditions leading to perfect business model innovation are self-efficacy and optimism in entrepreneurial PC and cognitive dimension in SC. Compared with configuration S5, we can find that in the core conditions, the cognitive dimension and relationship dimension of SC are interchanged, that is, with self-efficacy and optimism, the relationship network and common cognition represented by the cognitive dimension and relationship dimension can form a configuration with the core conditions to promote the innovation of perfect business model. Through the above analysis, this configuration is known as “cognitive-oriented.”

#### SE•HOPE•OPT•RD•SD

Configuration S7 shows that the core conditions leading to perfect business model innovation are self-efficacy, hope, optimism, and relationship dimension in SC, supplemented by structural dimension in SC. Since perfect business model innovation emphasizes market explicit demand and rapid market response, it emphasizes making up for the shortcomings of existing models through optimization, which not only needs the relationship and structure dimension in SC to provide sufficient resources for the innovation process, but also emphasizes maintaining optimism and self-confidence, forming synergy with SC by shaping the advantages of the internal model, and jointly promoting the innovation of perfect business model. Through the above analysis, this configuration is named “compound oriented type II.” In addition, by analyzing the results in Table 7, it can be seen that optimism in entrepreneurial PC is the core condition in the configurations S4–S7, which indicates that entrepreneurs need to look at the future business model innovation results with an optimistic attitude in the process of optimizing and adjusting the existing architecture and making an agile response to the market.

## Conclusion and Discussion

### Research Conclusion

**Figure 2:**
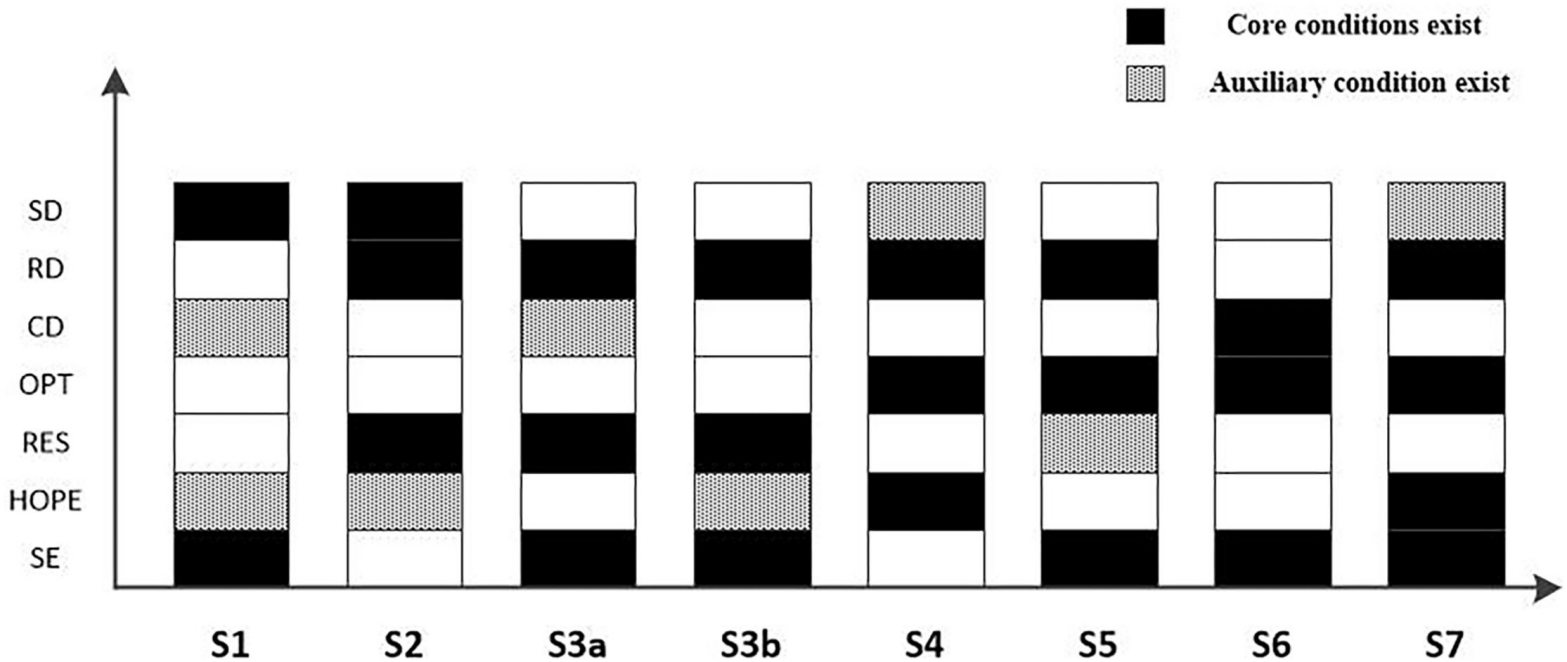
Pre-innovation configuration mode of business model of new ventures.

In this article, the resource conservation theory and signal theory are integrated, and based on the logic of “resource acquisition-resource utilization,” the influence path of different dimensions' configuration of entrepreneurial PC and SC on business model innovation of new ventures is explored. Through the research on survey data from entrepreneurs by using the fsQCA method, it shows that new ventures can choose different strategic configurations to better realize business model innovation based on different types of business model innovation, and the specific results are shown in Figure 2.

The antecedents and configurations of pioneering business model innovation can be divided into three models, namely, “belief-oriented,” “resilience-oriented,” and “compound-oriented I.” Among them, “belief-oriented” refers to the S1 configuration, which shows that pioneering business model innovation can be realized under the core conditions of self-efficacy and structural dimension; “Resilience-oriented” refers to the S2 configuration, which shows that pioneering business model innovation can be induced by the core conditions of resilience, relationship dimension, and structure dimension; “Composite-oriented type I” includes S3a and S3b, which indicates that pioneering business model innovation can be achieved under the joint promotion of self-efficacy, resilience, and relationship dimension.

The antecedents and configurations of perfect business model innovation can be divided into four models, namely, “hope-oriented,” “confidence-oriented', “cognition-oriented,” and “compound-oriented II.” Among them, “hope-oriented” refers to the S4 configuration, which shows that perfect business model innovation can be realized under the core conditions of hope, optimism, and relationship dimension; “Confidence-oriented” refers to the S5 configuration, which shows that the core conditions of self-efficacy, optimism, and relationship dimension can lead to perfect business model innovation; “Cognition-oriented” refers to the S6 configuration, which shows that perfect business model innovation can be achieved under the joint promotion of self-efficacy, optimism, and cognition; “Compound-oriented II” refers to the S7 configuration, which indicates that the innovation of perfect business model is realized under the joint promotion of various core conditions and auxiliary conditions. From the results shown in Figure 2, it can be seen that optimism constitutes a necessary condition among these four configuration conditions, and the perfect business model innovation cannot be separated from the optimistic psychological state of entrepreneurs.

### Theoretical Contribution and Managerial Implications

The theoretical contribution and managerial implications of this article are mainly manifested in the following three aspects.

First, this research enriches the research on the antecedents of business model innovation of new ventures. The existing research on the antecedents of business model innovation is mainly from the perspectives of new knowledge, resource actions, previous experience, and management ability (Zhou et al., 2022b), while the research on key resources, such as knowledge, experience, skills, and talents, is relatively few, and most of the existing research does not consider the types of business model innovation (Foss and Saebi, 2017; Clauss et al., 2019; Hock-Doepgen et al., 2021). On the basis of dividing the business model innovation of new ventures into pioneering innovation and perfect innovation, this article explores the influence mechanism of PC and SC on business model innovation from the research logic of “resource acquisition-resource utilization,” and the influence on the configuration of PC and SC with different dimensions and multiple concurrences on different types of business model innovation of new ventures, thus enriching and expanding the related research on the antecedents of business model innovation of new ventures.

Second, it expands the application of resource conservation theory and the application range of signal theory. The existing research on resource conservation theory mainly focuses on the field of “resources,” emphasizing and increasing the initial accumulation of resources and realizing the spiral of value-added resources (Hobfoll et al., 2018; Bickerton and Miner, 2021). This article applies the resource conservation theory to the field of entrepreneurship and proposes that new ventures can realize the interactive value-added of resources through business model innovation, thus expanding the application of resource conservation theory. In the field of entrepreneurship research, the related application exploration of signal theory mainly focuses on low-cost signals, such as entrepreneur's individual characteristics and entrepreneur's individual performance (Parhankangas and Ehrlich, 2014; LePine et al., 2016), but relatively few high-cost signals are involved. By introducing the high-cost signal of entrepreneurial SC, this article explores its role in the business model innovation of new ventures through the logic of “resource acquisition-resource utilization', thus expanding the application scope of signal theory.

Third, this article has inspired entrepreneurs and new enterprises that business model innovation is the result of the interaction and linkage value-added of internal and external resources. On the one hand, entrepreneurs should consciously improve their PC. Specifically, when new ventures tend to innovate pioneering business models, they should focus on improving the self-efficacy and resilience associated with “belief-oriented” and “resilience-oriented” configurations. When new ventures tend to carry out perfect business model innovation, they should focus on enhancing the hope and optimism related to the “hope-oriented” and “confidence-oriented” configurations. On the other hand, entrepreneurs should constantly improve the SC structure and send positive signals through the SC structure to provide external resources' support for the business model innovation of new ventures. In particular, entrepreneurs should combine the configuration that leads to the innovation of the business model, carry out the interaction of SC and PC in a targeted way, and realize the goals of the business model on the basis of capital appreciation.

### Research Deficiencies and Prospects

This article uses the questionnaire data to measure entrepreneurs' dual capital, and the accuracy of measurement needs to be improved. The better way to use fsQCA is to select case enterprises for data collection, which can be used in the future to obtain more accurate configuration data. Second, the internal and external resource elements that affect the business model innovation of new ventures include not only PC and SC but also human capital, relationship network, resource utilization mode, and other factors. In the future, researchers can consider using large sample data to add more antecedents to obtain a more practical and detailed business model innovation path. Finally, new ventures have different pursuits of value creation and value acquisition at different stages. In the future, longitudinal case data can be considered to obtain the data on different growth stages through long-term tracking, so as to further explore the antecedent configuration that changes with the growth of enterprises.

## Data Availability Statement

The raw data supporting the conclusions of this article will be made available by the authors, without undue reservation.

## Author Contributions

JZ is responsible for idea generation, manuscript writing for theoretical part, data collection, and responsible for data analysis. JZ and PY are responsible for idea generation and manuscript revision. YL and QC are responsible for the initial method part writing. All authors contributed to the article and approved the submitted version.

## Funding

This research was supported by the Natural Science Fund of Shandong Province of China (ZR2021MG043) and the Qingchuang Science and Technology Support Program of Shandong Province (2019RWG021).

## Conflict of Interest

The authors declare that the research was conducted in the absence of any commercial or financial relationships that could be construed as a potential conflict of interest.

## Publisher's Note

All claims expressed in this article are solely those of the authors and do not necessarily represent those of their affiliated organizations, or those of the publisher, the editors and the reviewers. Any product that may be evaluated in this article, or claim that may be made by its manufacturer, is not guaranteed or endorsed by the publisher.
